# Supplementary material for: Omega-3 Fatty Acids during Pregnancy in Indigenous Australian Women of the Gomeroi Gaaynggal Cohort
Source: Nutrients. 2023 Apr 18;15(8):1943. doi: 10.3390/nu15081943 (PMC10145055; doi:10.3390/nu15081943)
Supplement: Supplementary file 1 [file nutrients-15-01943-s001.zip › nutrients-2311402-supplementary.pdf]

nutrients MDPI

Supplementary Materials

Table S1. Supplemental fatty acid intake during pregnancy as recorded by 24-hr recall during the earlier stages of pregnancy.

	Uncomplicated (n = 63)	Preterm birth* (n= 10)	HDP* (n = 6)	Total (n = 75)
	Median (IQR)	Median (IQR)	Median (IQR)	Median (IQR)
**Nutrient**				
Total n-3 LC-PUFAs (mg/day)	0.0 (0.0 – 0.0)	0.0 (0.0 – 0.0)	0.0 (0.0 – 0.0)	0.0 (0.0 – 0.0)
EPA	0.0 (0.0 – 0.0)	0.0 (0.0 – 0.0)	0.0 (0.0 – 0.0)	0.0 (0.0 – 0.0)
DPA	0.0 (0.0 – 0.0)	0.0 (0.0 – 0.0)	0.0 (0.0 – 0.0)	0.0 (0.0 – 0.0)
DHA	0.0 (0.0 – 0.0)	0.0 (0.0 – 0.0)	0.0 (0.0 – 0.0)	0.0 (0.0 – 0.0)
ALA (18:3) (g/day)	0.0 (0.0 – 0.1)	0.0 (0.0 – 0.1)	0.0 (0.0 – 0.1)	0.0 (0.0 – 0.1)
LA (18:2) (g/day)	0.0 (0.0 – 0.1)	0.0 (0.0 – 0.1)	0.0 (0.0 – 0.1)	0.0 (0.0 – 0.1)
Total fat (g/day)	0.0 (0.0 – 1.0)	0.3 (0.0 – 1.5)	0.0 (0.0 – 1.0)	0.0 (0.0 – 1.5)

ALA: Alpha-linolenic acid. DHA: Docosahexaenoic acid. DPA: Docosapentaenoic acid. EPA: Eicosapentaenoic acid. HDP: Hypertension During Pregnancy. IQR: Interquartile range. LA: Linoleic acid. n-3 LC-PUFAs: Omega-3 long-chain polyunsaturated fatty acids. Uncomplicated: Those without preterm birth or HDP. *Four women had both a preterm birth and HDP.

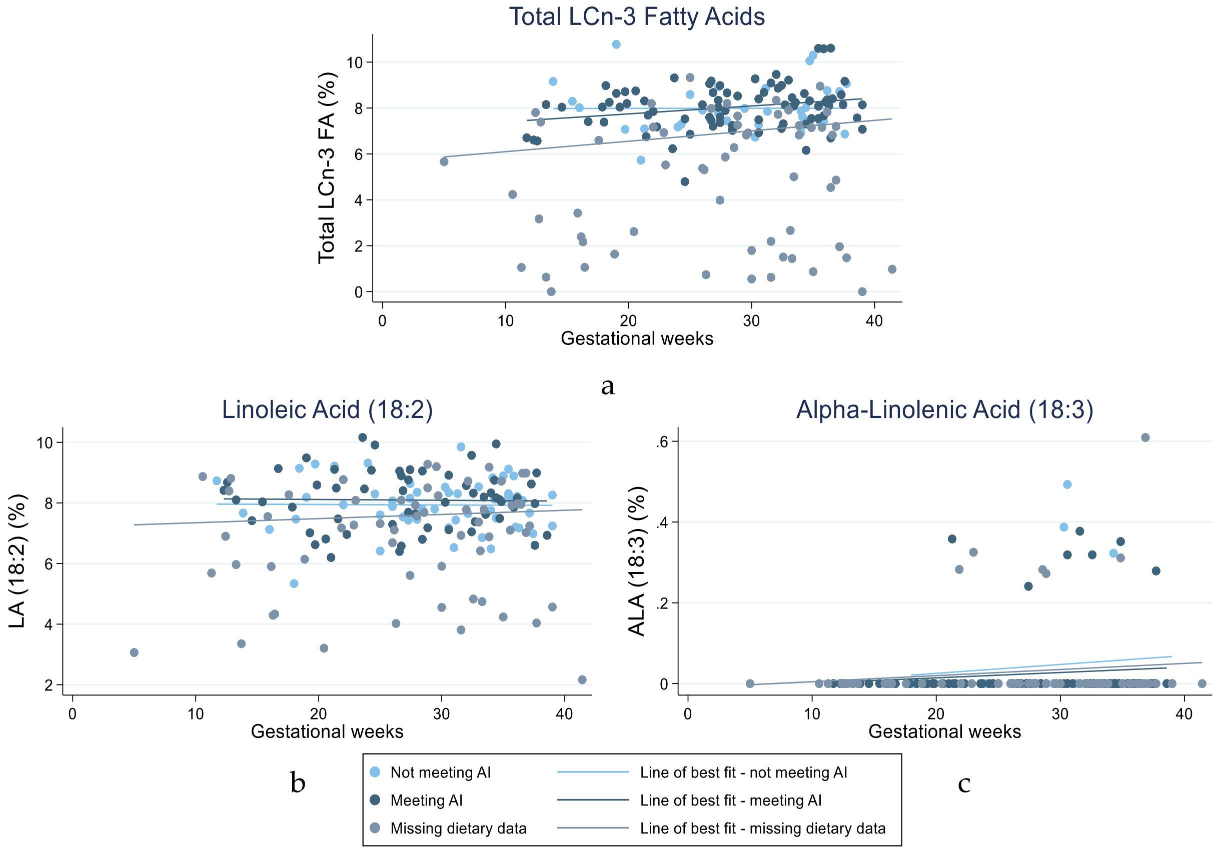

Figure S1. Fraction of LC-PUFAs in red blood cell membranes in pregnant women of the Gomeroi Gaaynggal cohort and those meeting national nutrient reference value guidelines: (a) Fraction of total n-3 LC-PUFAs (EPA, DPA and DHA) (**b**) Fraction of linoleic acid (18:2); (**c**) Fraction of alpha-linolenic acid (18:3).
